# S3 guideline diagnostics and therapy of alopecia areata – Part 1: Diagnostics and epidemiology

**DOI:** 10.1111/ddg.70065x

**Published:** 2026-03-19

**Authors:** Ulrike Blume‐Peytavi, Andria Constantinou, Tsenka Tomova‐Simitchieva, Adrian Tanew, Michael Sticherling, Hermann Girschick, Annika Vogt, Uwe Schwichtenberg, Uwe Gieler, André Märtens, Kerstin Zienert, Claudia Stenders, Ricardo Niklas Werner, Doris Wilborn

**Affiliations:** ^1^ Department of Dermatology, Venereology and Allergology Charité – Universitätsmedizin Berlin Corporate Member of Freie Universität Berlin and Humboldt‐Universität zu Berlin, Berlin Institute of Health Berlin Germany; ^2^ Private practice Vienna Austria; ^3^ Department of Dermatology University Hospital Erlangen Erlangen Germany; ^4^ Department of Pediatrics and Adolescent Medicine Vivantes Netzwerk für Gesundheit GmbH, Klinikum im Friedrichshain Berlin Germany; ^5^ Derma Nord Dermatology Practice Bremen Germany; ^6^ Psychodermatology Competence Center University Hospital Giessen, Vitos Giessen‐Marburg gemeinnützige GmbH, Vitos Psychosomatik Giessen Giessen Germany; ^7^ Hairdresser Berlin Germany; ^8^ Alopecia Areata Deutschland e.V. Krefeld Germany; ^9^ Department of Dermatology, Venereology and Allergology Division of Evidence‐Based Medicine (dEBM) Charité – Universitätsmedizin Berlin Corporate Member of Freie Universität Berlin and Humboldt‐Universität zu Berlin, Berlin Institute of Health Berlin Germany

**Keywords:** Alopecia areata, comorbidities, diagnosis, epidemiology, prognostic factors, risk factors

## Abstract

In the project funded by the Innovation Committee at the G‐BA, the S3 guideline for the diagnosis and treatment of AA was developed between 2023 and 2025. The interdisciplinary expert panel consisted of representatives from the German Dermatological Society, in particular from the Pediatric Dermatology Working Group, the Professional Association of German Dermatologists, the German Society for Psychosomatic Medicine and Psychotherapy, the German Society for Pediatrics and Adolescent Medicine, the Austrian Society for Dermatology and Venereology, a hairdresser as an external consultant and two patient representatives.

In the first of two parts, we present the key statements on epidemiology and the recommendations for diagnosis. Data from Germany show a population‐based prevalence of AA of 0.22% (95% CI 0.21–0.22) for 2016 and 0.21% (95% CI 0.20–0.22) for 2020. This means that 170,000 people in Germany were diagnosed with alopecia areata (AA) in 2020. International data show a pooled prevalence of 2.1%, but with large regional differences. Data on the incidence of AA from Germany show that 72 patients per 100,000 were newly diagnosed with AA in 2020 (RR 0.072%; 95% CI 0.07–0.08). Extrapolated to the German population, this means that around 70,000 people were newly diagnosed with AA in 2020.

## INTRODUCTION

This first part of the publication presents recommendations and excerpts from the background texts of the S3 guideline *Diagnostics and therapy of alopecia areata* on the diagnosis including epidemiology, comorbid diseases, prognostic factors and quality of life of alopecia areata (AA) before therapy. This first part is based on the full version dated September 18, 2025, which was developed in accordance with the *Association of the Scientific Medical Societies in Germany* (AWMF) version 2.0 (2020),^1^ and version 2.1 (2023),^2^ from January 2023 to October 2025. The second part contains the recommendations and background texts of the chapters on therapy, quality of life during therapy, psychosocial support and cosmetic services and should be used together with the first part of the guideline on diagnostics and epidemiology. The guideline targets patients with AA in all age groups, and the recommendations have been formulated specifically for children, adolescents, and adults.

Alopecia areata of the alopecia areata circumscripta (AC) type, which is the most common initial manifestation of AA, and the rare type of alopecia areata totalis (AT, ORPHA: 700), and alopecia areata universalis (AU, ORPHA:701) often occurs in childhood and adolescence and can be acute or chronic and recurrent, leading to complete loss of hair on the head, face, and body. In addition to functional limitations (eye inflammation, increased UV exposure), visible hair loss often leads to an enormous emotional and psychosocial burden that should not be underestimated. It often leads to social stigmatization, bullying, social withdrawal and a lasting reduction in quality of life. Sixty‐six percent of all AA patients experience the first manifestation of their disease in childhood and adolescence by the end of the third decade of life. Children, adolescents and young adults are often permanently impaired in their family and career development during the formative socialization phase. Evidence‐based diagnostic and therapeutic recommendations for the various forms of AA are generally lacking in Germany but are urgently needed specifically for this age group with special needs.

The project on which this publication is based was funded by the Innovation Committee of the Federal Joint Committee under the funding code 01VSF22016 (S3 LL AA) in the period from January 1, 2023 to June 30, 2025.

## METHODS

The methodological steps of guideline development were described in detail in the guideline report (see also the online supplement and the AWMF website https://register.awmf.org/de/leitlinien/detail/013‐104). In brief, some key details: At the kick‐off meeting in February 2023, the guideline group adopted the 45 key questions guiding the systematic literature search. It was determined that the focus of the literature search should be on epidemiological and therapeutic questions. Based on the results of the literature search, assessment and summary, the guideline group was able to adopt 79 recommendations for the entire guideline at the consensus conference in September 2024.

### Consensus process

The guideline group used the framework of the *Grading of Recommendations Assessment, Development and Evaluation (GRADE) Working Group*,^3^ see Table 1: Wording in the recommendations, as a basis for the uniform formulation of the recommendations. For easy recognition of the recommendations, they were inserted into separate boxes that followed a uniform structure: in the left‐hand column appears the serial number of the recommendation; the middle column shows the recommendation text using the standardized terms. Below this, the strength of consensus in the guideline committee and the evidence base including literature references (consensus‐based/evidence‐based) appear. In the right‐hand column, arrows and a colored background indicate the direction and strength of the recommendation. The classification of the strength of consensus can be found in Table 2.

**TABLE 1:** Scheme for grading recommendations.

Recommendation strength	Wording	Symbol	Interpretation
Strong recommendation for the use of an intervention	“We recommend…” Or “should”	**↑↑**	We believe that all or almost all informed people would decide in favour of this intervention. Clinicians need to spend less time on the decision‐making process with patients and can instead spend this time on overcoming barriers to implementation and treatment adherence. In most clinical situations, the recommendation can be adopted as a general approach.
Weak recommendation for the use of an intervention	“We suggest…” Or “ought to”	**↑**	We believe that most informed people, but a substantial proportion would not, would decide in favour of this intervention. Clinicians and other healthcare providers healthcare providers need to take more time in the decision‐making process with patients. Decision‐making processes in the healthcare system require in‐depth discussion and the involvement of many stakeholders.
Recommendation open / no recommendation regarding an intervention	“We cannot make a recommendation with respect to…” Or “can”	**0**	At present, a recommendation for or against this intervention cannot be made due to certain circumstances (e.g., unclear or balanced risk‐benefit ratio, no available evidence)
Weak recommendation against the use of an intervention	“We suggest against…” Or “should not”	**↓**	We believe that most informed people, but a substantial proportion would not, would decide against this intervention.
Strong recommendation against the use of an intervention	“We recommend against…” Or “shall not”	**↓↓**	We believe that all or almost all informed people would decide against this intervention. In most clinical situations, the recommendation can be adopted as a general approach.

**TABLE 2:** Classification of consensus strength.

Classification of consensus strength	
Strong consensus	> 95% of those entitled to vote
Consensus	>75–95% of those entitled to vote
Majority approval	>50–75% of those entitled to vote
No majority approval	<50% of those entitled to vote

All members of the guideline group received the complete background text in preparation for the preliminary online vote on the recommendations. The Delphi process offered the opportunity to vote on and comment on the draft recommendations submitted. The recommendations with more than 95% agreement were read out at the consensus conference and were considered to have been voted on. The recommendations that were still open were discussed and finally voted on. The conference, which took place as a hybrid event, was moderated by PD Dr. Ricardo Werner.

### External review/approval by the professional associations/implementation

The long version, the patient version and the algorithms of the guideline were submitted to the German Dermatological Society (DDG) as the lead specialist society and the other participating specialist societies for comment and approval. All feedback and how it was dealt with was recorded in the guideline report. The guideline will be implemented by publication on the websites of the AWMF, the DDG and the other participating specialist societies and the patient special interest group Alopecia Areata e. V.

### Updating and validity

The guideline is valid for five years (until September 17, 2030) and is to be updated after this period in the event of changes to key topics.

## DEFINITION OF ALOPECIA AREATA

Alopecia areata is a chronic, immune‐mediated disease characterized by acute onset, non‐scarring hair loss ranging from small, patchy hairless areas on the scalp to complete loss of scalp and body hair. According to current knowledge, it is assumed that the breakdown of the immune privilege (IP) of the hair follicle (HF), together with genetic and external factors, is primarily responsible for the manifestation of the disease.^4^

Alopecia areata can be associated with a considerable reduction in quality of life and significant psychosocial and somatic comorbidities.

**Table:** 

	**Statement**	
**S1**	AA is an autoimmune disease that can be associated with a considerable reduction in quality of life and significant psychosocial and somatic comorbidities.	
	Strong consensus	**Consensus‐based**

## EPIDEMIOLOGY

### Prevalence

Excerpts from the long version of the guideline are presented here, in particular the epidemiological data from Germany; further international data are presented in the long version.

Data on the population‐based prevalence of AA from Germany show the following results: Augustin et al. 2024 analysed data from a representative 40% sample of all adults who were insured with a German statutory health insurance company (DAK‐Gesundheit) between 2016 and 2020 (n  =  2.88 million). Based on at least one relevant outpatient or inpatient diagnosis of the *International Classification of Diseases* (ICD)‐10 L63 (L63, L 63.0, L 63.1, L 63.2, L 63.8, L63.9), the annual AA prevalence and incidence (ICD‐10 L63) and also the frequency of comorbidities for 2016 to 2020 were calculated.^5^

They calculated a population‐based prevalence of AA of 0.22% (95% CI 0.21–0.22) for 2016 and 0.21% (95% CI 0.20–0.22) for 2020. This means that 210 people per 100,000 were diagnosed with AA in 2020 (age‐ and gender‐standardized prevalence rate 0.21%). If extrapolated to the German population, this prevalence rate would mean that 170,000 people were diagnosed with AA in 2020. The authors also determined that the women included in the analysis from the age group > 40 years were more frequently affected than men (0.2% women compared to 0.1% men). In the age group 70 to < 80 years, the prevalence of AA in women was even more than 6.5 times higher than in men. In the age group < 40 years, men were slightly more frequently affected than women. Higher prevalence and incidence rates for AA were found in Hesse (prevalence 215 per 100,000; incidence 73 per 100,000), North Rhine‐Westphalia (prevalence 210 per 100,000; incidence 73 per 100,000) and Bremen (prevalence 235 per 100,000; incidence 62 per 100,000). However, the prevalence rates also tended to be higher in Berlin and Brandenburg (both prevalence 211 per 100,000). The lowest prevalence rates were found in Thuringia, Hamburg and Mecklenburg‐Western Pomerania.^5^

### Incidence

Data on the incidence of AA from Germany show that 72 patients per 100,000 were newly diagnosed with AA in 2020 (RR 0.072%; 95% CI 0.07–0.08). Extrapolated to the German population, this means that around 70,000 people were newly diagnosed with AA in 2020.^5^

## RISK FACTORS

Risk factors in medicine are understood to be “precursors and predictors of diseases and health disorders. A risk factor provides information about a potential health hazard that manifests itself directly or indirectly and usually only after a time lag.”^6^

Certain diseases, in particular other autoimmune diseases, are frequently observed in connection with AA. Some of these diseases are comorbidities in the sense of concomitant diseases occurring at the same time. Results from epidemiological studies show whether diseases are explicitly considered risk factors for AA and can therefore be differentiated from comorbidities. Case‐control studies (retrospective) and two arm‐cohort studies (prospective) are particularly suitable for this purpose. These studies compare whether people with a certain disease were more frequently exposed to a certain disease in the past than people without this disease. The significance of the respective factor is calculated using the odds ratio (OR) or hazard ratio (HR).

The following factors were agreed by 50 international dermatology experts as risk factors for AA: positive family history of AA or autoimmune disease, personal history of autoimmune disease, thyroid disease, vitiligo, atopy and/or atopic dermatitis.^7^

According to Meah et al. (2020), iron deficiency, pregnancy and vaccinations have no influence on the development of AA. Environmental factors, including bacterial or viral infections, trauma and acute stress, can act as trigger factors for the development of AA.^7^

Table 3 summarises the results of the included studies on risk factors. Risk factors for which study results showed a positive correlation are presented, as are study results that indicate no correlation. There are conflicting results regarding vitamin D deficiency, so vitamin D deficiency is not considered a risk factor based on the current study situation. The evidence on thyroid disorders is also contradictory: when all thyroid disorders are analysed together, a positive correlation is found, but subgroup analyses sometimes show no correlation, for example for hypothyroidism and hyperthyroidism.

**TABLE 3:** Effect estimates of risk factors for AA.

Risk factor	Outcome	Positive (References)	Negative (References)
Afro‐American (comparison with Whites)^8^	AA	OR 1,77 (95% CI 1,37–2,28)^8^	
Asian (comparison with Whites)^8^	AA		OR 0,40 (95% CI 0,32–0,50)^8^
Thyroid disease	AA	OR 1,91 (95% CI 1,23–2,94)^9^	
Hypothyroidism	AA	HR 1,88 (95% CI 1,30–2,71)^10^	OR 1,28 (95% CI 0,98–1,67)^9^
Hyperthyroidism	AA	aHR 9,29 (95% CI 7,11–12,14)^11^	OR 1,22 (95% CI 0,59–2,52)^9^
Graves Disease	AA	aHR 8,66 (95% CI 6,03–12,42)^11^	
Thyroiditis	AA	aHR 6,42 (95% CI 3,15–13,11)^11^	
Hashimoto Thyroiditis	AA		aHR 2,70 (95% CI 0,75–9,70)^11^
Vitiligo	AA	HR 3,13 (95% CI 1,08–9,10)^10^	
Systemic lupus erythematodes	AA	HR 5,43 (95% CI 2,11–13,97)^10^	
Rheumatoid arthritis	AA	HR 1,66 (95% CI 1,09–2,52)^10^	
Psoriasis	AA	HR 2,01 (95% CI 1,00–4,03)^10^	
Atopic dermatitis	AA	OR 9,72 (95% CI 4,38–21,59)^12^ RR 2,98 (95% CI 1,36–6,53)^12^ OR 2,36 (95% CI 1,52–3,67)^13^ OR 2,79 (95% CI 1,27–6,13)^14^	
Atopic dermatitis	AU	OR 2,98 (95% CI 1,95–4,55)^13^	
Metabolic syndrome: hyperlipidemia	AA		aHR 0,71 (95% CI 0,42–1,19)^15^
Metabolic syndrome: obesity	AA		aHR 1,01 (95% CI 0,42–2,43)^15^
Vitamin‐D‐deficit	AA	OR 2,3 (95% CI 2,2–3,1)^16^	HR 1,08 (95% CI 0,68–1,73)^17^
Bacterial infection	AA	OR 1,8 (95% CI 1,56–2,08)^18^	
Viral infection	AA	OR 2,0 (95% CI 1,67–2,38)^18^	
Helicobacter pylori‐infection	AA	OR 1,57 (95% CI 1,19–2,05)^19^	
Hepatitis‐C‐infection	AA	aHR 6,69 (95% CI 4,28–10,44)^20^	
Human Papilloma Virus (HPV)	AA	aHR 2,55 (95% CI 1,88–3,47)^21^	
Depression	AA	aHR 11,61 (95% CI 9,92–13,59)^22^ aHR 1,90 (95% CI 1,67–2,15)^23^ RR 1,66 (95% CI 1,24–2,2)^24^ OR 2,71; (95% CI 1,52–4,82)^25^	

*Abbr*.: AA, alopecia areata; aHR, adjusted hazard ratio; AU, alopecia universalis; CI, confidence interval; HR, hazard ratio; HPV, human papilloma virus; OR, odds ratio; RR, risk ratio

## ETIOPATHOGENESIS AND FORMS OF AA

For the contents of this chapter, please refer to the long version.

## DIAGNOSTICS AND DIFFERENTIAL DIAGNOSTICS

**FIGURE 1:**
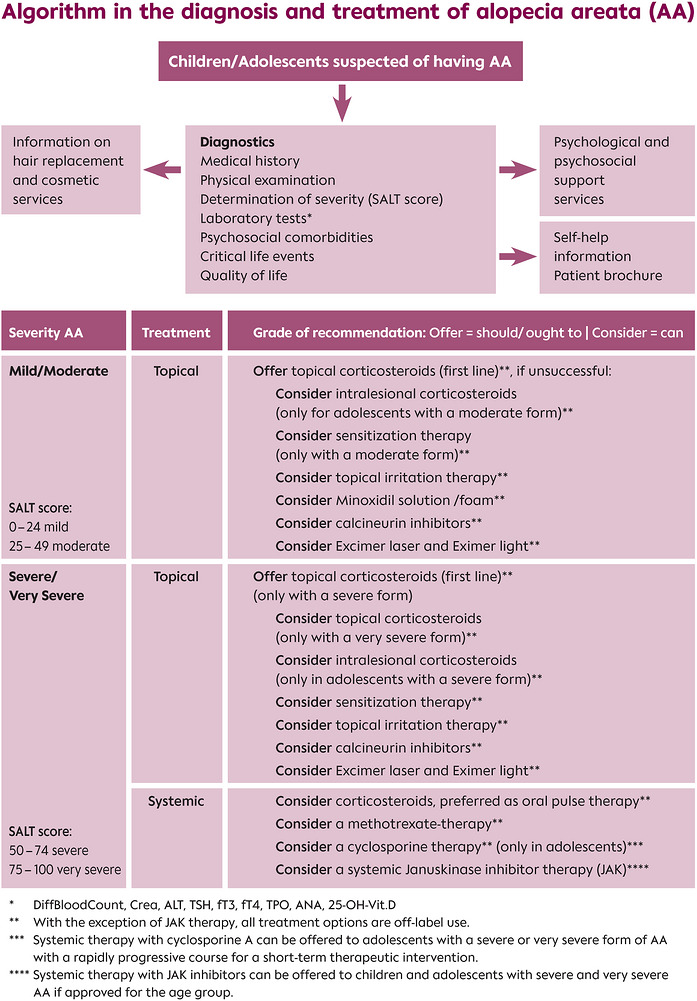
Algorithm for children and adolescents.

**FIGURE 2:**
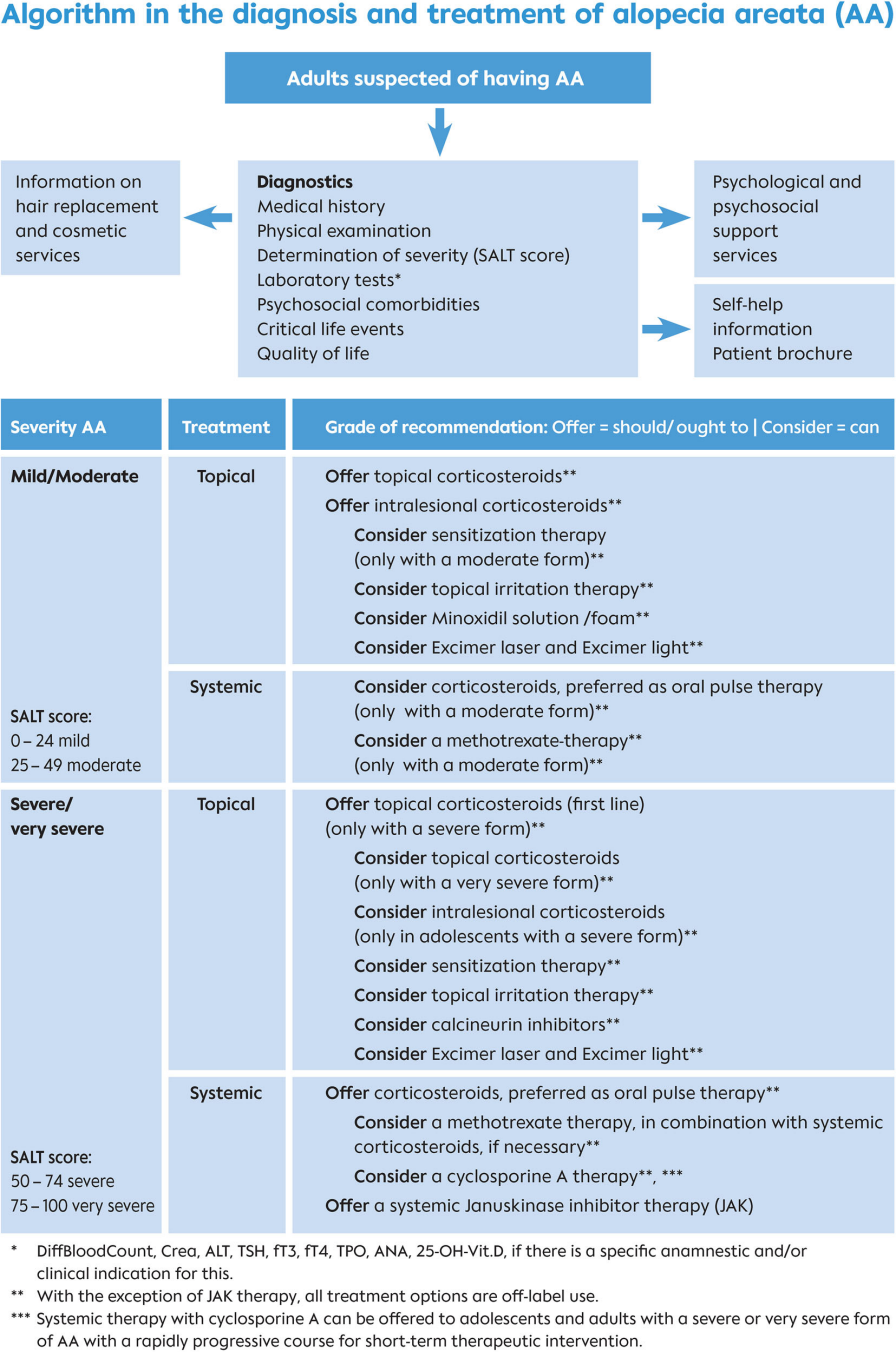
Algorithm for adults.

The following Figure 1 shows the algorithm for children and adolescents, while Figure 2 summarizes the algorithm for adults. The diagnostic recommendations are then presented. The numbering of the recommendations corresponds to the numbering in the long version.

### Medical history

**Table:** 

	Recommendation	Recommendation strength
**E10**	For the medical history, the age at first manifestation, the course of the disease, the current disease activity including previous relapses, the duration of the current and past relapses and associated symptoms *should* be asked.	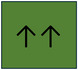
	Strong consensus	**Consensus‐based**

**Table:** 

	Recommendation	Recommendation strength
**E11**	The medical history *should* include other autoimmune or inflammatory diseases such as atopy, various autoimmune comorbidities (e.g., thyroid disease, vitiligo, inflammatory bowel disease), recurrent infections or inflammatory foci.	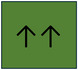
	Strong consensus	**Consensus‐based**

**Table:** 

	Recommendation	Recommendation strength
**E12**	The medical history *should* also include a positive family history of AA or other autoimmune diseases.	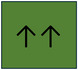
	Strong consensus	**Consensus‐based**

**Table:** 

	Recommendation	Recommendation strength
**E13**	The medical history *should* also include questions about previous treatments for AA and the response to these.	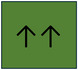
	Strong consensus	**Consensus‐based**

**Table:** 

	Recommendation	Recommendation strength
**E14**	A temporal connection between critical life events and the triggering of AA *ought to* be asked about in medical history and the significance critically assessed.	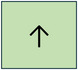
	Strong consensus	**Consensus‐based**

The special medical history for AA includes the age at first manifestation for stratification into age groups with different prognoses (e.g., before or after puberty, young adults, before/after menopause/adrenopause), the course of the disease (acute, intermittent, chronic), current disease activity, recording of previous relapses, the duration of the current and past relapses and the associated symptoms such as discomfort, itching and others. In addition, other autoimmune or inflammatory diseases such as atopic diathesis, various autoimmune comorbidities (e.g., thyroid disease, vitiligo, inflammatory bowel disease), recurrent infections or acute and/or chronic inflammatory foci, as well as current or recent topical or systemic therapies, should be assessed as part of the medical history. A family history of AA and autoimmune diseases is also requested at the initial presentation, as a positive family history of AA and other autoimmune diseases is relevant for the consultation.^26^ In addition, information on past critical life events in the last three months should be requested. Life events can be defined as events that happen to most people at some point in their lives, but which nevertheless represent an exceptional event for the individual.^27^ Life events include bereavement, the birth of a child, new job or marriage.^28^ Data from surveys of AA patients show that 24% of AA patients reported critical life events,^29^ or occur more frequently compared to healthy controls.^30^, ^31^

### Physical examination

**Table:** 

	Recommendation	Recommendation strength
**E15**	As part of a physical examination, a macroscopic inspection of the scalp and the entire integument, in particular the hair‐bearing areas and the nails, as well as a determination of the extent and pattern of hair loss *should* be carried out.	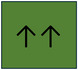
	Strong consensus	**Consensus‐based**

**Table:** 

	Recommendation	Recommendation strength
**E16**	As part of a physical examination, the appearance of the scalp and the skin of the integument within the hairless areas *should* be examined for signs of scarring, scaling, erythematous papules, pustules or crusts to rule out other differential diagnoses.	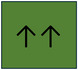
	Strong consensus	**Consensus‐based**

A thorough clinical examination includes a macroscopic inspection of the scalp and the entire integument, in particular the body hair and nails, as well as a determination of the extent and pattern of hair loss. In particular, the hair on the arms and legs is also evaluated. The whole‐body examination procedure requires a careful and cautious approach. Although the pattern of circumscribed hair loss of AA is usually characteristic, in rarer cases, such as diffuse alopecia areata (DAA) or alopecia areata incognita (AAI), a diffuse reduction in hair density is observed. In addition, the appearance of the scalp and the skin within the hairless areas is examined for signs of scarring, scaling, erythematous papules, pustules or crusts to rule out other differential diagnoses.^26^

### Diagnostic methods

**Table:** 

	Recommendation	Recommendation strength
**E17**	To diagnose AA, a dermoscopic examination of the alopecic areas *ought to* be performed on every patient with suspected AA.	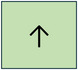
	Strong consensus	**Consensus‐based**

**Table:** 

	Recommendation	Recommendation strength
**E18**	A dermoscopic examination of the alopecic areas *ought to* be performed to monitor the progress of AA.	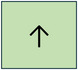
	Strong consensus	**Consensus‐based**

### Dermoscopic examination

Dermoscopic examination of the scalp, hair follicles and hair shafts, also known as trichoscopy, is a simple, non‐invasive microscopic examination that is very helpful in the diagnosis and follow‐up of scalp and hair diseases.^32^, ^33^, ^34^ The dermoscopic examination is particularly important for differentiating between non‐scarring and scarring hair diseases, especially for confirming the suspected clinical diagnosis of AA and evaluating its disease activity. In general, a dry dermoscopic examination is recommended first to avoid overlooking scales or perifollicular keratoses, followed by application of immersion oil or disinfectant spray; this can be dispensed with in dermatoscopes with polarized light. The most common dermoscopic findings in AA are yellow dots, black dots, broken hairs, exclamation mark hairs and vellus hairs, while other less frequently described findings are vertical hairs, vertical dots and black dots, less frequently described findings, upright hairs, tapered hairs, pigtail hairs and Pohl‐Pinkus constrictions are.^35^, ^36^, ^37^, ^38^ Detailed descriptions of the individual findings are summarized in Table 4. Yellow dots occur mainly in long‐standing lesions and in more severe forms of AA, AT and AU. Although yellow dots are a typical finding in AA, they can also occur in other hair disorders such as androgenetic alopecia (AGA) or trichotillomania.^34^ Black dots and short, broken hairs are typical findings in the active border area of AA foci, but both can also be observed in trichotillomania. Exclamation mark hairs are pathognomonic for AA and are typically found at the active margin of AA foci. Vellus hairs in areas typically characterized by intermediate or terminal hairs are associated with remission or long‐standing disease.^38^

**TABLE 4:** Characteristic dermoscopic findings in AA (from Lintzeri et al.^26^).

Dermoscopic findings in AA	Description
Yellow dots	Round, yellow or pinkish‐yellow circular dots representing the dilated but intact hair follicle openings filled with sebum or remnants of keratinocytes
Black dots	Remains of hair shafts that have broken off in the hair canal, especially in dark‐haired patients with a light skin type
Broken hair	Short, broken hair shafts
Exclamation mark hair	Short, broken hairs that taper towards their proximal end
Vellus hair	Thin, unpigmented downy hairs
Vertical hair	Healthy, vertically regrowing hair with a tapered distal end; also found in telogen effluvium, trichotillomania, tinea capitis and temporal triangular alopecia
Pointed hair	Normal‐looking hairs with a pointed proximal end; precursor of exclamation mark hairs and black dots
Pigtail hairs	Short, regrowing, curled hairs with tapered ends; indicate a remission of the loss episode
Pohl‐Pinkus constrictions	Progressive and irregular thinning along the hair shaft; an indication of the severity of the disease; also found in chemotherapy‐induced alopecia

### Hair pluck test

**Table:** 

	Recommendation	Recommendation strength
**E19**	For the differential diagnosis and for the assessment of disease activity at baseline and during the course of the disease, a hair plucking test *ought to* be performed on the edge of the lesions and the contralateral clinically unaffected side in every patient with suspected AA.	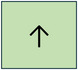
	Strong consensus	**Consensus‐based**

A hair plucking test is helpful for the differential diagnosis and to determine the disease activity. A tuft of 50–60 hairs is firmly grasped close to the scalp and pulled moderately in the direction of growth, at the edge of the individual lesions and on the contralateral, clinically unaffected side.^39^, ^40^ Washing or brushing the hair does not affect the validity of the hair pluck test.^41^ A positive hair plucking test with epilation of ≥ 10% of the hairs recorded indicates active disease, while a negative test with less than two hairs plucked out indicates “normal” hair loss.^41^ A positive plucking test in a clinically unrecognizable affected area may indicate progressive disease with a diffuse course.^42^

### Trichogram

**Table:** 

	Recommendation	Recommendation strength
**E20**	A trichogram *can* be carried out in individual cases for diagnostic purposes, especially for differential diagnosis.	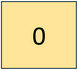
	Strong consensus	**Consensus‐based**

The use of a trichogram in the diagnosis of AA is internationally controversial. This is a microscopic hair root examination after epilation and embedding of 60–80 hairs, which are removed five days after the last hair wash using a clamp and a practiced plucking technique.

Rudnicka and colleagues consider the trichogram to be a useful complementary tool for clinical assessment, diagnosis and monitoring response to treatment.^33^ Another expert panel rated the trichogram as not helpful for diagnosis. The experts also disagreed on the evaluation of the trichogram for the assessment of disease activity.^32^ At best, the trichogram can distinguish a DAA from a telogen effluvium, as a DAA is characterized by the presence of anagen‐dysplastic and dystrophic hairs.^43^ However, it should be borne in mind that a predominance of telogen hairs due to the inflammatory invasion of hair follicles with increased induction of the telogen phase can also be observed in the chronic stage of AA.

### Biopsy

**Table:** 

	Recommendation	Recommendation strength
**E21**	A biopsy of the scalp *ought to* be performed if diffuse AA is suspected or if the clinical picture is not clear to rule out other differential diagnoses.	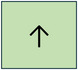
	Strong consensus	**Consensus‐based**

**Table:** 

	Recommendation	Recommendation strength
**E22**	If a biopsy is to be performed to diagnose AA, it *should* be carried out at the edge of the circumscribed focus, avoiding a site typical of androgenetic alopecia.	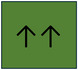
	Strong consensus	**Consensus‐based**

In an international expert consensus, all participants agreed that skin biopsies can be considered in selected situations if the clinical findings of AA are inconclusive in order to rule out other diseases.^33^

A scalp biopsy is particularly indicated if scarring alopecia cannot be clinically excluded with certainty, if a single lesion is resistant to treatment or in the case of DAA for differential diagnostic clarification.^32^ In the context of AA, a single biopsy is usually sufficient to establish the diagnosis. The biopsy is performed at the edge of the circumscribed focus, avoiding a site typical of androgenetic alopecia.^32^ An additional scalp biopsy from non‐lesional scalp is not considered important.^32^ The histopathologic findings in AA depend on the activity of the disease at the time of biopsy. In the acute (early) stage, the main feature in the findings is a peribulbar and intrabulbar lymphocytic infiltrate surrounding the anagen or catagen follicles, described as a “swarm of bees”. The infiltrate consists mainly of CD4^+^ and CD8^+^ T cells; however, eosinophils, mast cells and plasma cells can also be detected. In addition, a premature or increased percentage transition to the catagen or telogen phase is observed. In long‐term persistent (chronic) AA, the intensity of infiltration may vary; most HFs are in the telogen phase, and miniaturized HFs may also be present. However, an increased number of empty HFs can also be observed. In some cases, keratin plugs in empty follicular ostia show a long‐lasting course without regrowth at.^44^, ^45^

### Differential diagnosis

**Table:** 

	Recommendation	Recommendation strength
**E23**	If the clinical findings of AA are not clear, a careful differential diagnosis *should* be carried out depending on the clinical findings and the age group.	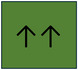
	Strong consensus	**Consensus‐based**

The differential diagnosis (DD) of AA primarily includes diseases that are associated with non‐scarring, circumscribed or diffuse hair loss. Depending on the age group, different DDs must be considered: Trichotillomania, tinea capitis and temporal triangular alopecia are the main clinical pictures that should be considered when differentiating circumscribed AA, especially in childhood and adolescence, while telogen effluvium, AGA with a female pattern of hair loss and drug‐induced alopecia are mainly considered for DD in diffuse forms of AA. The entire spectrum of DDs of AA is summarized in Table 5.^26^

**TABLE 5:** Common differential diagnoses (DD) of circumscribed and diffuse alopecia areata, grouped by age (children/adolescents and adults).

	DD patchy AA	DD diffuse AA
Children	Tinea capitis	Loose or short anagen hair syndrome
	Trichotillomania	Telogen effluvium
	temporal triangular alopecia (Nevus Brauer)	Congenital hypotrichosis
Adolescents/adults	Follicular mucinosis, folliculotropic mycosis fungoides	Telogen effluvium
	Alopecia syphilitica	AGA with female hair loss pattern
	Scarring alopecia such as chronic discoid lupus erythematosus (CDLE), lichen planopilaris	Drug‐induced (antiproliferative) alopecia

Abbr.: AA, alopecia areata; AGA, androgenetic alopecia; DD, differential diagnosis

### Classification tools

**Table:** 

	Recommendation	Recommendation strength
**E24**	The SALT score *ought to* be used to determine the severity of AA at initial presentation and for follow‐up assessment.	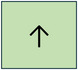
	Strong consensus	**Consensus‐based**

**Table:** 

	Recommendation	Recommendation strength
**E25**	The use of the ClinRo instrument or PRO scales for eyelashes and eyebrows *can* be considered to determine the severity of involvement of the eyebrows, eyelashes and nails.	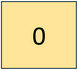
	Strong consensus	**Consensus‐based**

The assessment of the severity of AA is of great importance for patient counselling and treatment planning. The severity of AA guides the physician in therapeutic decision‐making and helps to assess the response to therapy and the prognosis of the disease. To facilitate and standardize the assessment of the severity and progression of AA in clinical studies, a simple but reliable and reproducible severity score was developed: the so‐called *Severity of Alopecia Tool (SALT) score*.^46^ The SALT score is used in clinical practice as a measure for assessing the extent of AA in the scalp.^32^ The scalp is divided into four quadrants, each representing a percentage (%) of the total scalp area: left side (18%), right side (18%), top (40%) and back (24%). To determine the SALT score, which has a maximum of 100%, the percentage of hair loss in each quadrant is visually estimated and then added together. Based on the SALT score calculated in this way, five degrees of severity of hair loss can be distinguished: S0  =  no hair loss, S1 < 25% hair loss, S2  =  25%–49% hair loss, S3  =  50%–74% hair loss, S4  =  75 %–99% hair loss, S5  =  100% hair loss.^46^, ^47^ Usually, a SALT ≥ 50 % is considered severe AA and a SALT ≥ 75 % is considered very severe AA. One limitation of the SALT score is that it does not consider the possible involvement of other anatomical areas, which are important for assessing the severity of the disease and prognosis. In order to take into account the manifestations of AA outside the scalp, further assessment scales are available to supplement the SALT score. For example, eyebrow, eyelash, beard, armpit, pubic hair and other involvement can be assessed to improve the characterization of individual patients.^48^ This method takes into account the extent of scalp involvement (SALT score), the pattern of scalp AA, the number of other affected anatomical sites and their extent. Similarly, Jang et al. (2016) developed the *Alopecia Areata Progression Index (AAPI)* to assess the overall activity of hair loss in AA patients with pigmented hair by adding clinical findings related to hair loss (plucking test and dermoscopic examination findings) to the SALT score and thus an extended score can be calculated.^49^ Instruments such as the ClinRo instrument and other patient‐oriented assessment tools (PRO) are available to assess the severity of eyebrow, eyelash and nail involvement.^50^ Ideally, the assessment is accompanied by photo documentation.^50^

In summary, in daily clinical practice, calculation of the SALT score, identification of other affected anatomical sites and inspection of the nails for AA‐related lesions are essential elements in the assessment of disease severity.

### Laboratory values

**Table:** 

	Recommendation	Recommendation strength
**E26**	If AA is present, screening for thyroid disease *ought to* be carried out.	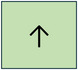
	Strong consensus	**Consensus‐based**

**Table:** 

	Recommendation	Recommendation strength
**E27**	In adults with AA, blood tests (differential blood count, kidney and liver function), thyroid values (TSH, TPO, ± TRAK, ±TG), ANA titre and 25‐OH‐Vit. D *ought to* be carried out if there is a specific anamnestic and/or clinical indication for this.	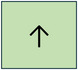
	Strong consensus	**Consensus‐based**

**Table:** 

	Recommendation	Recommendation strength
**E28**	In children and adolescents with AA, blood tests (differential blood count, kidney and liver function), thyroid values (TSH, fT3, fT4, TPO), ANA titer and 25‐OH‐vitamin D *ought to* be examined to screen for possible comorbidities.	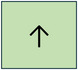
	Consensus	**Consensus‐based**

**Table:** 

	Recommendation	Recommendation strength
**E29**	If there are anamnestic and/or clinical indications of the presence of other autoimmune diseases, targeted laboratory diagnostics *ought to* be carried out.	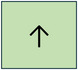
	Strong consensus	**Consensus‐based**

**Table:** 

	Recommendation	Recommendation strength
**E30**	An infectious serological examination, in particular with regard to virological triggers, *shall not* be determined if there is no additional suspicion of infection based on anamnestic and clinical findings.	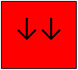
	Strong consensus	**Consensus‐based**

**Table:** 

	Recommendation	Recommendation strength
**E31**	Before initiating systemic treatment of AA, the same (laboratory) tests *should be* carried out for the medication to be used as for other dermatological diseases, in accordance with the technical information and general recommendations for action for the respective active substance.	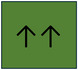
	Strong consensus	**Consensus‐based**

To date, there are no validated biomarkers that can be determined in the diagnosis of AA.^33^ There is international disagreement on several laboratory parameters, such as whether routine screening for vitamin D deficiency and thyroid disease should be performed.^32^ Nevertheless, the guideline group decided in favour of the recommendation formulated above. In addition, the following tests are limited to individual cases only, they are not recommended for all patients diagnosed with AA and, above all, are only recommended for differential diagnostic clarification:

Complete blood count, kidney and liver function, screening for other autoimmune diseases, connective tissue diseases, celiac disease, pernicious anaemia and diabetes.

In the absence of relevant clinical symptoms and signs, infectious serologic testing for viruses is not useful to identify a potential trigger for an AA episode. Morning cortisol levels are not a useful test in patients who believe that stress may have triggered an AA episode. Before initiating systemic treatment of AA, the same investigations are required as for the use of the respective therapeutic agents in other dermatological diseases.^32^

### Mycology

**Table:** 

	Recommendation	Recommendation strength
**E32**	A mycological examination *should* be carried out if there is a clinical suspicion of tinea capitis.	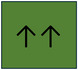
	Strong consensus	**Consensus‐based**

A mycological diagnosis is not a routine examination. It is only necessary if there is a clinical suspicion of tinea capitis.

### Comorbidities and associated autoimmune diseases

**Table:** 

	Recommendation	Recommendation strength
**E33**	Psychosocial comorbidities, especially anxiety (social anxiety) and depression, as well as stigmatization *ought to* be taken into account in the diagnosis.	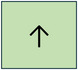
	Strong consensus	**Consensus‐based**

AA can occur together with various other disorders. Table 6 summarizes the detailed description of the literature on comorbidities from the long version:

**TABLE 6:** Frequency of comorbidities in AA.

Comorbidity	Chance	Reference
Vitamin‐D‐deficit^1^	65.4% (95% CI 50.6–77.8)^*^	^51^
Helicobacter pylori‐Infectionen^1^	62.8% (95% CI 46.2–76.9)^*^	
Psychiatric diseases^1^	49.2% (95% CI 17.8–81.5)^*^	
Anxiety^1^	27.1% (95% CI 17.7–39.2)^*^	
Depression^1^	18.9% (95% CI10.9–30.8)^*^	
Atopic diseases^1^	20.6% (95% CI 16.2–25.9)^*^	
Allergic Rhinitis^1^	17.7% (95% CI 14.1–21.9)^*^	
Atopic Dermatitis^1^	9.6% (95% CI 6.2–14.4)^*^	
Thyroid disorders^1^	8.0% (95% CI 5.9–10.7)^*^	
Hyperinsulinemia^2^	60.8% (95% CI 46.9–73.1)*	
Alexithymia^2^	52.9% (95% CI 37.1–68.2)*	
Metabolic syndrome^2^	37.3% (95% CI 25.2–51.2)*	
Lens changes^2^	32.1% (95% CI 18.7–57.8)*	
Retinal changes	24.0% (95% CI 13.8–38.5)*	
Audiologic changes^2^	17.3% (95% CI 0.5–89.4)*	
Atopic dermatitis	RR 2,9; 95% CI 2,7–3,2^**^	^5^
Pruritus	RR 2,7; 95% CI 2,4–3,1^**^	
Lupus erythematodes	RR 2,4; 95% CI 1,7–3^**^	
Urticaria	RR 2,3; 95% CI 1,9–2,7^**^	
Anxiety	8%^***^	^52^
Atopic dermatitis	5%^***^	
Autoimmune thyroid disease	4%^***^	

^1^ Results are based on several studies with a high total number of cases.

^2^ Results are based on very small case numbers from a few studies.

* Prevalence is based on at least one study with a comparison group of healthy individuals, relative to individuals with AA.

** With a prevalence of <3% of the total population.

*** Prevalence in relation to AA patients with co‐morbidity.

### PROGNOSTIC FACTORS

Prognostic factors predict the future course of a disease.^53^ At this point, we refer to all factors that can influence the further course of the disease, the severity of AA and the response to treatment as prognostic factors. These are particularly important for understanding and planning the treatment concept and for patient counselling.

A detailed description of the literature was provided in the long version of the guideline; only the prognostic factors identified by the literature search and their significance are shown here in Tables 7 and 8.

**TABLE 7:** Prognostic significance.

Outcome	Prognostic factor	Study results or consensus
Progressive course of disease	Positive personal history for autoimmune diseases	Consensus
	Positive personal history for atopy	Consensus
	Duration of the disease over five years	Consensus
	Ophiasis type	Consensus
	Nail participation	Consensus
	Loss of eyelashes and other body hair	Consensus
Response to therapy	Age at first manifestation	Consensus
	History of autoimmune or atopic disease	Study results^54^, ^55^, ^56^, ^57^, ^58^, ^59^, ^60^
	Nail participation^*^	Study results^57^
Relapse	Family history of an AA	Study results^61^
	Duration of illness > 6 months	Study results^54^, ^62^

* Nail involvement: conflicting results, therefore listed in Tables 7 and 8.

**TABLE 8:** No prognostic significance.

Outcome	Prognostic factor	Study results or consensus
Response to therapy	Iron deficiency	Consensus
	Vaccination	Consensus
	Hypothyroidism (children)	Study results^56^
	Atopic dermatitis (children)	Study results^56^
	Atopic diathesis (children)	Study results^56^
	Duration of illness > 1 Jahr	Study results^57^
	Nail participation^*^	Study results^54^, ^58^, ^60^
	Age at first manifestation	Study results^54^, ^55^, ^63^
	Family history of an AA	Study results^54^, ^55^, ^56^, ^58^, ^59^, ^60^, ^61^, ^64^
Progressive course	Positive family history of an organ‐specific autoimmune or atopic disease	Consensus
	Initiation of AA therapy in the first six months	Consensus

* Nail involvement: conflicting results, therefore listed in Tables 7 and 8.

### QUALITY OF LIFE AT THE TIME OF DIAGNOSIS

The effects of hair loss can go far beyond purely optical concerns for each individual affected,^65^ which can often have psychological and psychosocial consequences. These include a diminished sense of personal attractiveness, lower self‐confidence and self‐esteem, and negative effects on social interactions.^66^ Analysis of the quality of life of people with AA shows how they perceive their physical, mental and social health in relation to their illness.^67^ The chronic or often relapsing course of AA contributes significantly to the impairment of patients' quality of life.

Various instruments are available to measure quality of life, from generic instruments to dermatology‐specific instruments such as the Dermatology Quality of Life Index (DLQI).^68^ The overall values of the DLQI can be interpreted as follows: 0–1  =  no impact on the patient's life, 2–5  =  low impact, 6–10  =  moderate impact, 11–20  =  very high impact, 21–30 extremely high impact on the patient's life.^68^

In a meta‐analysis, data on the DLQI score of 3978 adults with AA and an average age of 23 to 51 years were pooled from 14 studies. The pooled value of the DLQI was 6.67 (95% CI 5.54–7.81). This score is considered a moderate impairment of quality of life. The authors of the meta‐analysis found a high degree of heterogeneity in the results of the included studies, with average DLQI scores ranging from 2.1 (95% CI 1.91–2.29) to 10.6 (95% CI 9.53–11.85). The meta‐analysis leaves open the extent to which the severity of AA, the type of therapy or the duration of the disease influenced the quality of life.^69^

However, data from an American cross‐sectional study showed correlations with the severity of AA, eyebrow loss and quality of life. The study analysed data from 259 patients who had a mild form of AA (average SALT score 12.8). The Skindex 16 questionnaire was used as an instrument to measure quality of life. It showed an average quality of life of 37.1 (SD 23.6) points with a possible maximum value of 100 points; a higher value means a higher impairment of quality of life. The authors used regression analysis to calculate additional factors that predict impairment in quality of life. The result showed that the severity of AA, eye irritation and loss of eyebrows predicted a higher impairment in quality of life (moderate severity: 12.9; 95% CI 6.1–19.6); severe severity: 14.9; 95% CI 6.7–23.2; eye irritation: 37.0; 95% CI 7.1–66.9; Eyebrow hair loss: 6.8; 95% CI –0.5 to 14.0, whereby the result for the eye irritation factor is associated with great uncertainty, only two patients in the study reported eye irritation. Duration of disease had no predictive value (0.8; 95% CI 0.3–1.3), and men were more likely to have a higher quality of life compared to women (–10.7; 95% CI –16.0 to –5.4).^70^

AWMF register number: 013–104

## CONFLICT OF INTEREST STATEMENT

A complete list of declared conflicts of interest can be found in the guideline report on the AWMF website: https://register.awmf.org/de/leitlinien/detail/013‐104.
